# Massachusetts Veterinary Association

**Published:** 1891-01

**Authors:** Austin Peters

**Affiliations:** Secretary


The meeting then adjourned.

D. S. Breslin, D.V.S.,

*Secretary.*

---

*Massachusetts Veterinary Association.*—The regular meeting was held at No. 19 Boylston Place, Wednesday evening, November 26th, 1890.

Presiding officer, President Thomas Blackwood. Members present: Drs. Blackwood, Emerson, Ferguson, Hadcock, Howard, Marshall, Winchester, Billings and the Secretary. Honorary member, Dr. Stickney. Essayist, Dr. E. C. Becket.

Minutes of last meeting read and accepted. There was no new business. The next in order was the reading of a paper by Dr. Becket upon "Surgical Treatment of Recto-Vaginal Fistula." The paper consisted chiefly of the notes of two cases of recto-vaginal fistula treated at the Harvard Veterinary Hospital, on Village Street. The essayist said that the cause was generally parturition, and was often complicated with rupture of the perineum, including a portion of the sphincter ani. He then described the two cases referred to above. Both were complicated with tear of the perineum, although one was much worse than the other. They were much benefited by treatment, the fistula being closed; but it was impossible to bring about repair of the sphincter ani in either case. Consequently, in going down hill the gut would fill with air until they had a bloated appearance, and this would pass off on level ground, together with more wind, rendering them disagreeable to drive. The treatment consisted in freshening the edges of the opening between the rectum and vagina, and bringing them together with sutures, silver wire being mostly used. It was found best to operate by throwing the mare and then etherizing. The animal, when under the influence of ether, relaxed the parts, and thus rendered them more accessible. The intestines should be as empty as possible before operating, and kept quiet as long as possible afterward, to avoid disturbance of the parts by the passage of feces. Both cases required to be operated upon two or three times before success was attained, and then it was only partial on account of the torn sphincters. The essayist thought that in cases of recto-vaginal fistula, where the perineum or sphincter was not injured, that treatment would be attended with success.

The ensuing discussion followed the reading of Dr. Becket's paper.

Dr. Winchester said that he had seen a few cases of recto-vaginal fistula, but all were in mares worth from $2.50 to $10.00, generally in the hands of cheap traders. He had tried operating, but with very little success. He spoke of Becket, in his paper, writing that the wall of the fistula was treated as one tissue, while anatomically it was two. Why would it not be better to try and separate the two walls, and sew each one by itself? Dr. Winchester appreciates the difficulty of doing this, but why would it not be doing the work more correctly to sew the rectal and vaginal walls separately?

Dr. Howard thought that in an old lesion it would be almost impossible to separate the walls of the intestine and vagina.

Dr. Marshall moved that the essayist be accorded a vote of thanks for his paper. Seconded; carried.

Moved by Dr. Winchester and seconded by Dr. Hadcock, that the secretary cast one ballot for Dr. Becket's admission as a member of the Association. Carried. Dr. Becket is accordingly elected.

Dr. Ferguson spoke of a case of deafness in a horse caused by firing a carbine close to his head. Blistering around the base of the ears was first resorted to without success, then treatmeut by electricity was tried with successful results in the course of a week.

Dr. Hadcock reported a case of rabies in a horse belonging to the West End Street Railway Co. In August last, a dog ran into a blacksmith's shop at Mt. Auburn, and bit a dog and the horse. Both dogs were killed at the time, and the horse worked as usual within a few days, when he began to show symptoms of rabies, soon becoming very violent and dying in less than twenty-four hours of the time when the first symptoms were noticed. The horse died in just three months and sixteen days from the time he was bitten. The brain and a part of the spinal cord were removed and sent to Dr. Jackson, at the Harvard Medical School, for the purpose of inoculating some rabbits in order to confirm the diagnosis of rabies. A general discussion of rabies followed, in which Drs. Marshall, Stickney, Winchester, Ferguson and Billings took part. Dr. Billings said that he was very skeptical about many cases we call rabies being rabies, and said that he did not believe in the rabies of Pasteur. He said that he had no faith in Pasteur after he persisted in keeping the "Newark children" among his statistics of patients treated after being bitten by a rabid animal, when he had been informed that the dog which bit them never had rabies. He then spoke of his rabies among cattle in the West, in which he separated a germ that would again produce the disease, but it was not altogether like rabies after all. Dr. Billings will not believe in the work done on rabies until the disease is produced by inoculation in dogs, and the dogs produce the disease in other dogs by biting them. He is also very skeptical as to the value of inoculation against a disease which has always been fatal, with no history of recovery and non-recurrence.

Dr. Winchester was appointed a committee of one to attend to inviting Dr. Van Schaick, of the Pasteur Institute in New York, to be present at one of the meetings of this association this winter, and give an address upon "Rabies."

A motion was made, seconded and carried, that we omit the December meeting, as the fourth Wednesday in December this year would bring the meeting on Christmas Eve.

Meeting then adjourned. AUSTIN PETERS, *Secretary.*

